# Anthocyanin Accumulation in the Leaves of the Purple Sweet Potato (*Ipomoea batatas* L.) Cultivars

**DOI:** 10.3390/molecules24203743

**Published:** 2019-10-17

**Authors:** GuoLiang Li, Zhaomiao Lin, Hong Zhang, Zhonghua Liu, Yongqing Xu, Guochun Xu, Huawei Li, Rongchang Ji, Wenbin Luo, Yongxiang Qiu, Sixin Qiu, Hao Tang

**Affiliations:** Institute of Crop Sciences, Fujian Academy of Agricultural Sciences, Scientific Observing and Experimental Station of Tuber and Root Crops in South China, Ministry of Agriculture. Fuzhou, Fujian 350013, China; uslgl@126.com (G.L.); linzhaomiao@foxmail.com (Z.L.); teeteeking@163.com (H.Z.); lhl8620@163.com (Z.L.); qingqing0722@126.com (Y.X.); xgc_faas@163.com (G.X.); fjpotato@126.com (H.L.); jrc1976@163.com (R.J.); lwb9630@163.com (W.L.); qyxlm@sohu.com (Y.Q.); tanghao9403@163.com (H.T.)

**Keywords:** sweet potato, anthocyanin compositions, biosynthesis structural genes, transcription factor

## Abstract

Sweet potato anthocyanins are water-soluble pigments with many physiological functions. Previous research on anthocyanin accumulation in sweet potato has focused on the roots, but the accumulation progress in the leaves is still unclear. Two purple sweet potato cultivars (Fushu No. 23 and Fushu No. 317) with large quantities of anthocyanin in the leaves were investigated. Anthocyanin composition and content were assessed with ultra-performance liquid chromatography diode-array detection (UPLC-DAD) and ultra-performance liquid chromatography/quadrupole time-of-flight mass spectrometry (UPLC-QTOF-MS), and the expressions of genes were detected by qRT-PCR. The two cultivars contained nine cyanidin anthocyanins and nine peonidin anthocyanins with an acylation modification. The acylation modification of anthocyanins in sweet potato leaves primarily included caffeoyl, *p*-coumaryl, feruloyl, and *p*-hydroxy benzoyl. We identified three anthocyanin compounds in sweet potato leaves for the first time: cyanidin 3-*p*-coumarylsophoroside-5-glucoside, peonidin 3-*p*-coumarylsophoroside-5-glucoside, and cyanidin 3-caffeoyl-*p*-coumarylsophoroside-5-glucoside. The anthocyanidin biosynthesis downstream structural genes *DFR4*, *F3H1*, anthocyanin synthase (*ANS*), and UDP-glucose flavonoid 3-O-glucosyltransferase (*UFGT3*), as well as the transcription factor *MYB1*, were found to be vital regulatory genes during the accumulation of anthocyanins in sweet potato leaves. The composition of anthocyanins (nine cyanidin-based anthocyanins and nine peonidin-based anthocyanins) in all sweet potato leaves were the same, but the quantity of anthocyanins in leaves of sweet potato varied by cultivar and differed from anthocyanin levels in the roots of sweet potatoes. The anthocyanidin biosynthesis structural genes and transcription factor together regulated and controlled the anthocyandin biosynthesis in sweet potato leaves.

## 1. Introduction

**Figure 1:**
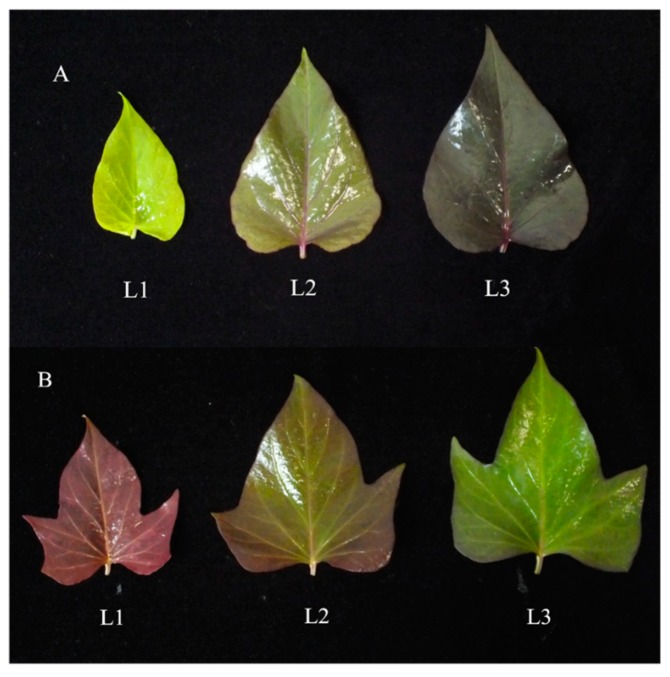
Photographs of sweet potato leaves with differential levels of accumulated anthocyanins. (**A**) Fushu No. 23, (**B**) Fushu No. 317, “L1” indicates the primary leaves (three days old), “L2” indicates the second leaves (five days old), and “L3” indicates the third leaves (seven days old).

The sweet potato (*Ipomoea batatas* L.) is an important tropical crop, providing starch, beta-carotene, and anthocyanins for human nutrition and industrial use [1]. Leafy sweet potato is a new type in China that is consumed for its stems and leaves, not the tuberous root. The stems and leaves are nutritious and contain high levels of protein, dietary fiber, calcium, magnesium, iron, zinc, flavonoids, and anthocyanins [2]. In 2003, the first leafy sweet potato cultivar was cultivated in China, called Fushu No. 7-6 [3], but in 2016 there was only one purple leafy sweet potato cultivar being grown in China, Fushu No. 23. Fushu No. 23 has been found to have high levels of anthocyanin in both the stems and leaves (Figure 1).

Anthocyanins are a group of water-soluble natural pigments that are considered flavonoids with a basic structure of C6–C3–C6. They are responsible for fruit and flower coloration [4]. Previous research on anthocyanins in sweet potato has focused on the roots and found that the basic anthocyanin monomers are cyanidin, peonidin, and pelargonidin. These anthocyanin monomers are often linked to acylated glucoside or sophoroside, and acylation modification comes from caffeic acid, *p*-coumaric acid, ferulic acid, and *p*-hydroxybenzoic acid [4,5,6,7,8]. Terahara et al. (1999) identified eight acylated anthocyanins in the roots of the purple Japanese sweet potato cultivar Yamagawamurasaki, and six monomers were identified as diacylated anthocyanins [9]. Tian et al. (2005) identified 26 anthocyanins from the ‘Ayamurasaki’ line that had been root cultured, and it was first discovered that the purple-fleshed sweet potato contained pelargonidin 3-sophoroside-5-glucoside and pelargonidin 3-feruloylsophoroside-5-glucoside [4]. Lee et al. (2013) separated six pelargonidin-based anthocyanins from Korean purple-fleshed sweet potato cultivar Borami [6], and found that compared with the purple-fleshed root, anthocyanins in sweet potato leaves were relatively less studied. Islam et al. (2002) detected 15 anthocyanin compounds from sweet potato leaves, and eight cyanidin derivatives were identified and measured [10]. Su et al. (2019) identified 14 anthocyanins with a new anthocyanin peonidin 3-caffeoyl-*p*-coumaryl sophoroside-5-glucoside in the leaves of three sweet potato varieties [11]. Vishnu et al. (2019) found that peonidin derivatives were the major anthocyanins in tubers and the leaves, but that the contents of the cyanidin derivatives were greater in leaves than in tubers [12]. Studies have shown that anthocyanins from sweet potato have good properties regarding scavenging 1,1-diphenyl-2-picrylhydrazyl (DPPH) radicals [13], and protect the liver [14,15], have anti-tumor properties [16], lower blood sugar levels [17], and have other positive physiological functions on the human body [18,19].

In this study, two purple sweet potato cultivars (Fushu No. 23 and Fushu No. 317) with different patterns of anthocyanin accumulation were studied (Figure 1). The content and chemical structures of anthocyanin monomers in the leaves were analyzed and the expression of anthocyanin biosynthetic structural genes and transcription factors were studied.

## 2. Materials and Methods

### 2.1. Chemicals

All of the reagents or solvents were of analytical, HPLC, or HPLC-MS grade. Absolute ethanol, acetone, hydrochloric acid (HCl), and sodium hydroxide (NaOH) were obtained from Sinopharm Chemical Reagent Co., Ltd. (Shanghai, China). Methanol and ethanol in LC-MS grade was purchased from Honeywell (Muskegon, MI, USA). Acetonitrile in LC-MS grade was obtained from Merck KgaA (Darmstadt, Germany). Formic acid in LC-MS grade was the product of Fisher Scientific (Waltham, MA, USA). The standard cyanidin 3-*O*-glucoside was purchased from Sigma-Aldrich (St. Louis, MO, USA). The TransStart Top Green qPCR SuperMix was purchased from Transgen Biotech (Beijing, China).

### 2.2. Plant Materials

The purple-leaf sweet potato cultivars Fushu No. 23 and Fushu No. 317 were sampled under natural outdoor light and temperature conditions. Two-thirds of samples were stored at −80 °C in a refrigerator (Haier Group Co., Qingdao, China) for later analyses of metabolites. To determine the content of total anthocyanins, the remaining 1/3 of leaves were oven-dried, ground into powder with a diameter less than 0.3 mm, and stored in a vacuum pack (VIP320, Beijing Torch SMT Inc. Co., Beijing, China) at 4 °C.

In order to understand the difference in anthocyanin levels between roots and leaves, some purple-fleshed sweet potatoes were examined, the cultivars Fushu No. 9, Fushu No. 24, Fushu No. 317, Ornamental Purple (OP), Fushu No. 23, and Fushu No. 25 were obtained from the Institute of Crop Sciences, Fujian Academy of Agricultural Sciences, Fuzhou, China, (26°08′ N, 119°28′ E) in August 2018. Yanshu No. 5, Longzishu No. 6, and Longzishu No. 8, were generously provided by the Longyan Institute of Agricultural Sciences, Longyan, China, in August 2018.

### 2.3. Sample Preparation

Two-thirds of lyophilized samples (50 mg) were grounded by TissueLyser JX-24 (Jingxin, Shanghai, China) with beads at 40 Hz for 4 min, and extracted with 0.5 mL of 70% methanol containing 0.1% formic acid. Samples were then processed for 10 min 100 HZ ultra-sonication in ice water. The mixtures were vortexed for 30 s and left to stand for 2 h at −40 °C. After, samples were centrifuged at 4 °C at 14,000 rpm for 15 min, and 350 μL of supernatant were dried under gentle nitrogen stream and re-dissolved in 90 μL of 70% methanol containing 0.1% formic acid combined with 10 μL of 25 μg/mL lidocaine (internal standard) prior to ultra performance liquid chromatography/quadrupole time-of-flight mass spectrometry (UPLC-QTOF-MS) analysis. Quality control (QC) sample was obtained by isometrically mixing the prepared samples. The injection volume was 1 μL (ESI^+^).

### 2.4. Ultra Performance Liquid Chromatography Diode-Array Detection (UPLC-DAD) and UPLC-QTOF-MS Analysis

Chromatographic separation was performed on an ACQUITY UPLC I-Class system (Waters Corporation, Milford, MA, USA) with an ACQUITY UPLC BEH C18 column (100 × 2.1 mm, 1.7 μm, Waters Corporation, Milford, MA, USA) maintained at 45 °C. The injection volume was 3 μL. The mobile phases consisted of water (phase A) and acetonitrile (phase B), both with 0.5% formic acid (*v/v*). A linear gradient elution was performed using the following program: 0–2 min, 1% B; 3 min, 5% B; 9 min, 20% B; 12 min, 50% B; 15 min, 100% B; 17 min, 100% B; 17.1 min, 1% B, and held for 20 min.

The eluents were analyzed on a Vion IMS QTOF Mass spectrometer (Waters Corporation, Milford, MA, USA) set to ESI^+^ mode. The capillary voltage was set to 2 kV. The sampling cone voltage and cone gas flow were 40 V and 50 L/h, respectively. The desolvation gas was maintained at a flow rate of 900 L/h and a temperature of 450 °C. The ion source temperature was 115 °C. The TOF-MS scan was operated at a high-resolution with 0.2 s survey scan time and a range of 50–1000 *m/z* in the continuum mode for both function 1 and 2. To improve the identification of unknown metabolites, MS^E^ function was also performed to obtain fragment ion information with a ramp collision energy from 20 to 45 eV. The mass accuracy calibration was performed with the 250 ng/mL lock mass leucine-enkephalin at 5 μL/min, with data acquisition frequency set at 30 s. The software for controlling the instrument and collecting data was UNIFI 1.8.1 (Waters Corporation, Milford, MA, USA). Peak picking, alignment, and deconvolution were conducted using Progenesis QI (Nonlinear Dynamics, Newcastle, UK) with default parameters. A suitable quality control sample (QC from pooled samples) from the run was selected as a reference for peak alignment. The structural identification of anthocyanins was performed using UNIFI 1.8.1(Waters Cooperation, Milford, MA, USA). The anthocyanin content was expressed as mg of cyanidin 3-*O*-glucoside equivalent.

### 2.5. Quantitative Real-time PCR ANALYSIS of Anthocyanin Biosynthetic Genes in Sweet Potato Leaves

Total RNA was isolated from sweet potato leaves in September using TransZol Plant (Transgen Biotech Inc., Beijing, China) following the manufacturer’s instructions. First-strand cDNA were reverse transcribed using a Reverse Transcriptase M-MLV Kit (Promega, Madison, WI, USA). The qRT-PCR assay was performed on an ABI QuantStudio5 Real-time system (ABI, Foster City, CA, USA), and in 10 μL of reaction containing 2× TransStart Top Green qPCR SuperMix (Transgen Biotech Inc., Beijing, China), 10 μM of solution from each primer (Supplementary Table S1), and 100 ng of cDNA. Thermocycling conditions were: initial denaturation at 95 °C for 2 min, followed by 40 cycles for 15 s at 95 °C, and 1 min at 60 °C. The relative expression of anthocyanin biosynthetic genes was assessed by the comparative threshold cycle (Ct) method [20]. The sweet potato actin gene (NCBI accession: EU250003) served as an internal control for signal normalization. Expression levels were evaluated as technical duplicates of biological triplicates from separate plant samples.

## 3. Results and Discussion

### 3.1. Total Monomeric Anthocyanin Content in the Leaves of the Cultivars Fushu No. 23 and Fushu No. 317

**Figure 2:**
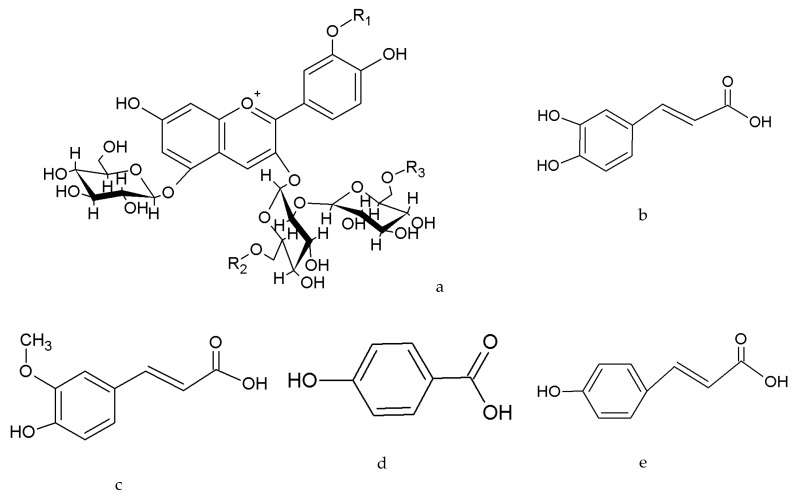
Skeleton structures of anthocyanins in sweet potato leaves. (**a**) Skeleton structures of anthocyanins; (**b**) caffeic acid (*m*/*z* 180); (**c**) ferulic acid (*m*/*z* 194); (**d**) *p*-hydro benzoic acid (*m*/*z* 138); and (**e**) *p*-coumaroyl acid (*m*/*z* 164).

To further understand the differences between the two purple sweet potato cultivars in the accumulation of anthocyanins, the composition and content of anthocyanins were detected by UPLC-DAD and UPLC-QTOF-MS (Figure 2, Table 1 and Table 2). Eighteen anthocyanin monomers were found, which contained a typical anthocyanin spectrum of a maximum absorbance at 520 nm.

The *m*/*z* ratio of the 18 anthocyanin monomers with daughter fragments was captured within the scanning interval range. Nine cyanidin was detected at *m*/*z* 287 and nine peonidin was detected at *m*/*z* 301. Cyanidin 3-sophoroside-5-glucoside (Table 1, Figure S1.1, *m*/*z* 773) produced three fragments located at *m*/*z* 611, 449, and 287. Transition 773 > 611 and 773 > 449 represented the loss of glucose (*m*/*z* 162) and sophorose (*m/z* 324), respectively, while transition 773 > 287 produced cyanidin (*m*/*z* 287) aglycone due to the loss of both glucose and sophoroside. Another example for mono- and di-acylated anthocyanin was cyanidin 3-(6’’-*p*-hydroxybenzoylsoph)-5-glucoside (Table 1, Figure S1.3, *m/z* 893). The transitions from 893 to 731 indicated a loss of glucose [M − 162]^+^, and from 893 to 449 [M − 162 × 2 − 120]^+^ indicated a loss of sophoroside and *p*-hydroxybenzoic acid 120 [*p*-hydroxybenzoic acid-H2O]^+^. The remaining anthocyanins were identified in a similar fashion using the LC-MS library and identification results of Tian and Lee et al. [4,6] The *m*/*z* ratios of each intact anthocyanin and its daughter fragments are summarized in Table 1. We identified four anthocyanin compounds that were found in the leaves of sweet potato for the first time: cyanidin 3-*p*-coumarylsophoroside-5-glucoside, peonidin 3-*p*-coumarylsophoroside-5-glucoside, cyanidin 3-caffeoyl-*p*-coumarylsophoroside-5-glucoside, and peonidin 3-caffeoyl-*p*-coumarylsophoroside-5-glucoside.

The composition of anthocyanins in the sweet potato leaves had certain regularity. The first anthocyanin monomers was cyanidin, another anthocyanin monomer peonidin was formed by methylation at the cyanidin R1 position, and other monoacylanthocyanins or diacylanthocyanins were formed by acylation modification at cyanidin or peonidin at the R2 and R3 positions [4,5,6,7]. The structure of the acylated anthocyanins was more stable and had stronger physiological activities than nonacylated anthocyanins. The acylation modification of anthocyanin in sweet potato leaves primarily included: caffeoyl, *p*-coumaroyl, feruloyl, and *p*-hydroxy benzoyl (Figure 2).

### 3.2. Differential Accumulation of Anthocyanins in the Leaves of the Cultivars Fushu No. 23 and Fushu No. 317

As shown in Figure 1, the anthocyanin accumulation in the leaves of the cultivars Fushu No. 23 and Fushu No. 317 was different. The anthocyanin content in Fushu No. 23 leaves increased with the growth of the first to third leaves (one- to seven-day-old from new leaves), while the anthocyanin content in Fushu No. 317 leaves decreased in that period.

The total anthocyanin content in sweet potato leaves ranged from 4.28 to 127.33 μg/g fresh weight (FW). The accumulation of anthocyanin monomers in the leaves of Fushu No. 23 and Fushu No. 317 was different (Table 2). In Fushu No. 23, the simple structure of anthocyanidin monomers led cyanidin 3-sophoroside-5-glucoside to increase from the first to second leaves, and then decrease from the second to third leaves. Meanwhile, the structurally complex monomers in Fushu No. 317 caused cyanidin 3-(6’,6′’-dicaffeylsophoroside)-5-glucoside to increase from the first to the third leaves.

### 3.3. Expression of Anthocyanidin Biosynthesis Structural Genes and Transcription Factor

**Figure 3:**
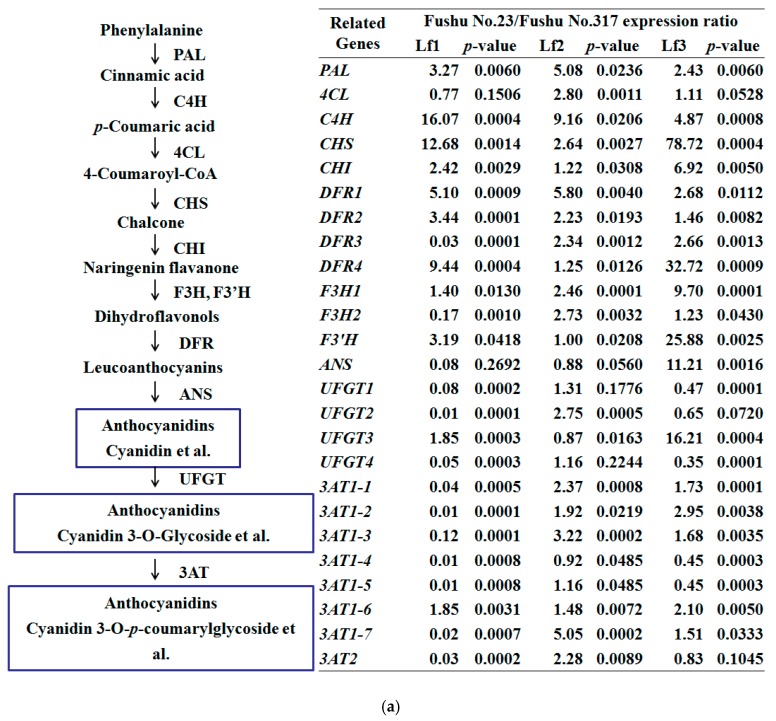

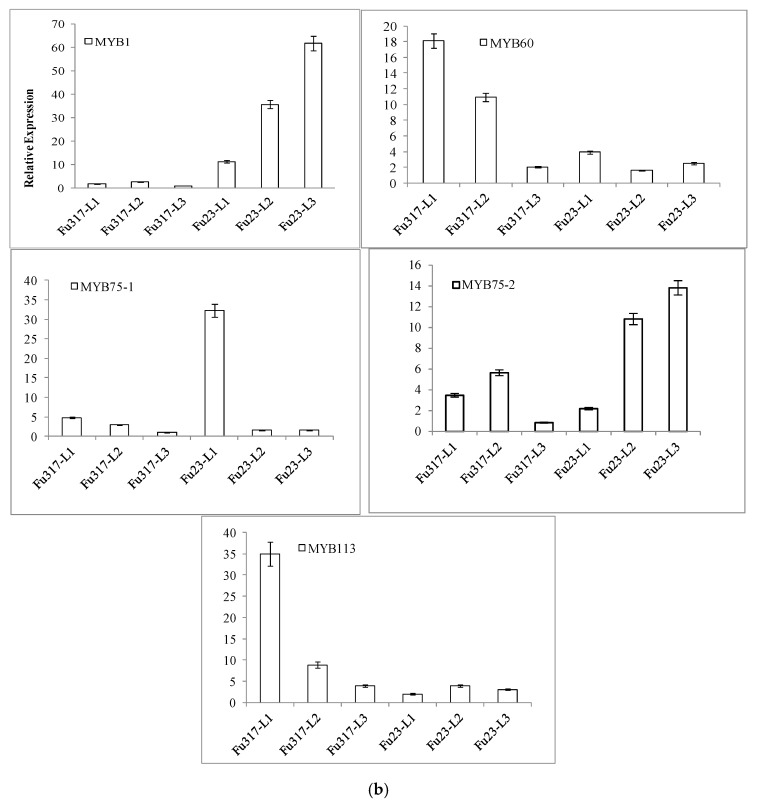
Expression of anthocyanin structural genes and transcription factors in the purple sweet potato cultivars Fushu No. 23 and Fushu No. 317. (**a**) Simplified model of the anthocyanin biosynthetic pathway and qRT-PCR analysis of the transcript abundance of the anthocyanin biosynthesis-related genes in Fushu No. 23 and Fushu No. 317 leaves. (**b**) Expression of transcription factors *IbMYB*s in sweet potato leaves.

The synthesis precursor of anthocyanin monomers in sweet potato leaves is phenylalanine, and structural genes in the synthesis process include: phenylalanine ammonialyase (*PAL*), cinnamic acid-4-hydroxylase (*C4L*), 4-coumarate:CoA ligase (*4CL*), chalconesynthase (*CHS*), chalconeisomerase (*CHI*), flavanone 3-hydroxylase (*F3H*), flavonoids 3′-hydroxylase (*F3′H*), dihydrofavonol4-reductase (*DFR*), and anthocyanin synthase (*ANS*) [21]. The first anthocyanin monomer was cyanidin, and other acylated anthocyanin monomers formed under the action of the UDP-glucose flavonoid 3-O-glucosyltransferase (*UFGT*) [22] or anthocyanin 3-O-acyltransferases (*3AT*) [23,24] genes (Figure 3). *DFRs*, *UFGTs*, and *3Ats* are mined from two diploid wild relatives of cultivated sweet potato (*Ipomoea triloba*. L). *UFGT1* (*3GGT*) was found to transfer glucose to glycosylated anthocyanins in purple sweet potato [25], but this is the only UFGT whose function in the sweet potato has been established. The transcription factors in anthocyanin synthesis were *MYB*, *bLHL*, and *WDR40* [21,26]. *IbMYB1* is a key regulatory gene of anthocyanin biosynthesis in the storage roots of purple-fleshed sweet potato [27]. *IbMYB60* is homologous to the *Arabidopsis* R2R3-MYB transcription factor *AtMYB60*, a transcriptional repressor of anthocyanin biosynthesis in lettuce [28]. *AtMYB75* (*PAP1*) [29,30] and *AtMYB113* (*PAP2*) [31,32] regulate the anthocyanin pathway in *Arabidopsis* seedlings. Two homologous *AtMYB75* genes (*IbMYB75-1* and *IbMYB75*-2) were mined from *Ipomoea triloba* genome, but only one homologous gene *IbMYB113* was found in the *Ipomoea triloba* genome (www.sweetpotato-garden.kazusa.or.jp).

To further clarify the internal connection of anthocyanin content with transcription levels of the genes involved in anthocyanin biosynthesis, 25 anthocyanin structural genes and five transcription factor genes were detected by quantitative real-time PCR. We found there were substantial differences in the expression of anthocyanidin-related genes in Fushu No. 23 and Fushu No. 317, and we found that the two cultivars had an opposite accumulation pattern. From the first to third leaves of Fushu No. 317, *CHS*, *DFR*s, *F3H*s, *ANS*, *UFGT*s, and *3At*s had a tendency to first increase and then decrease. Most anthocyanin biosynthesis related-myb transcription factors also had the same pattern. However, *DFR*s were correlated with the increase in anthocyanin content in the first to third leaves of Fushu No. 23.

On comparing the first leaf of Fushu No. 23 to Fushu No. 317, anthocyanin content was greater in Fushu No. 317 than Fushu No. 23, while the expression of *ANS* in the leaf of Fushu No. 23 was lower than in Fushu No. 317. Therefore, we speculate that *ANS* is the key gene for anthocyanin biosynthesis in the first leaf of sweet potato.

The anthocyanin content in the third leaf was also different. Expression of the structural genes: *CHS*, *DFR4*, *F3′H1*, *ANS,* and *UFGT3* were greater in Fushu No. 23 than in Fushu No. 317. Therefore, *CHS*, *DFR4*, *F3′H1*, *ANS*, and *UFGT3* may be vital regulatory genes of anthocyanin biosynthesis in sweet potato leaves.

MYB transcription factors (TFs) have had a long association with anthocyanin biosynthesis. In sweet potato, activation of anthocyanin levels by IbMYB1 was first observed in a functional complementation experiment using a mutant. Mano et al. (2007) reported *IbMYB1* was only expressed in the roots of purple-fleshed sweet potatoes but not in other related-tissues such as stems, leaves, flowers, and the roots of orange-, yellow-, or white-fleshed varieties [24], but we found *IbMYB1* can be highly expressed in the leaves of sweet potato variety Fushu No. 23, and can have very low expression in the leaves of Fushu No. 317. Perhaps the biosynthesis of anthocyanins in the leaves of sweet potato is through more than one metabolic pathway. *MYB60* and *MYB113* decreased from the first to third leaves, while *MYB1* and *MYB75-2* increased from the first to third leaves, possibly due to the fact that MYB has different effects on anthocyanin biosynthesis in different sweet potato leaves.

### 3.4. Anthocyanins in Roots of Purple-fleshed Sweet Potato Were Different from the Anthocyanins in Sweet Potato Leaves

In order to further determine differences in the composition of anthocyanins between sweet potato leaves and roots, we used the same method to analyze the anthocyanin components of five sweet potato leaves and five sweet potato roots. Fushu No. 317, Fushu No. 25, and OP contained 18 anthocyanin components and Fushu No. 23 and Yanshu No. 5 contained 17 anthocyanin components. However, cyanidin 3-*p*-coumarylsophoroside-5-glucoside and cyanidin 3-caffeoyl-*p*-coumaryl sophoroside-5-glucoside were not found in the any of the roots in this study. Interestingly, Tian et al. (2005) found cyanidin 3-*p*-coumarylsophoroside-5-glucoside and cyanidin 3-caffeoyl-*p*-coumaryl sophoroside-5-glucoside in a PL (purple line) cell line generated from the storage root of the purple-fleshed sweet potato cultivar Ayamurasaki [4], but this has not been identified in any earlier reports on purple-fleshed sweet potato [9,33]. It is possible that these compounds were not found in our study because they existed at such a low level that they could not be detected.

In this study, among five purple-fleshed sweet potatoes in this study, Longzishu No. 6 had the greatest number of anthocyanin monomers in the roots. The total contents of anthocyanins in Longzishu No. 6 roots were 255.75 ± 17.39 μg/g FW (Table 3), not very high. Anthocyanin monomers cyanidin 3-*p*-coumarylsophoroside-5-glucoside, cyanidin 3-caffeoyl-*p*-coumaryl sophoroside-5-glucoside, and cyanidin 3-caffeoyl-feruloylsophoroside-5-glucoside were not found in the roots of Fushu No. 317. The total contents of anthocyanins in Fushu No. 317 leaves (188.81 ± 18.35 μg/g FW) were much lower than that in the roots (1009.29 ± 66.41μg/g FW). As Su et al. speculated [11], maybe anthocyanin biosynthesis between sweet potato leaves and roots involved different phenotypes. Fushu No. 9 lacked two anthocyanin monomers: peonidin 3-*p*-coumarylsophroside-5-glucoside and peonidin 3-caffeoyl-*p*-coumarylsophoroside-5-glucoside in roots. Fushu No. 24 and Longzishu No. 4 only contained 13 anthocyanin monomers in the roots. It should be noted that the total anthocyanins in the roots of purple-fleshed sweet potato had greater changes with different varieties, but the total anthocyanins in the leaves of sweet potato showed small changes. A future study to explain the genotype of anthocyanin biosynthesis genes in the leaves and roots of sweet potato may be necessary.

## Figures and Tables

**Table 1:** Identification of anthocyanins (1–14) from cultivar Fushu No. 23 and cultivar Fushu No. 317 leaves using ultra performance liquid chromatography/quadrupole time-of-flight mass spectrometry (UPLC-QTOF-MS/MS).

Peak	Rt ^a^ (min)	Fragment Ions (*m*/*z*)			[M]^+^ (*m*/*z*)	Identity ^b^
1	4.45	287	449	611	773.2117	Cy 3-soph-5glc
2	4.99	301	463	625	787.2266	Peo 3-soph-5glc
3	5.92	287	449	731	893.2325	Cy 3-*p*-hydroxybenzoylsoph-5glc
4	6.10	287	449	773	935.2421	Cy 3-caffeylsophsoph-5glc
5	6.63	301	625	745	907.2479	Peo 3-*p*-hydroxybenzoylsoph-5glc
6	6.77	301	463	787	949.2578	Peo 3-caffeylsophsoph-5glc
7	6.86	287	449	757	919.2477	Cy 3-*p*-coumarylsoph-5glc
8	6.98	287	449	787	949.2577	Cy 3-feruloylsoph-5glc
9	7.50	301	463	771	933.2634	Peo 3-*p*-coumarylsoph-5glc
10	7.64	301	463	801	963.2734	Peo 3-feruloylsoph-5glc
11a	7.83	287	449	935	1097.2741	Cy 3-dicaffeylsoph-5glc
11b	7.89	287	449	893	1055.2636	Cy 3-caffeoyl-*p*-hydroxybenzoylsoph-5glc
12a	8.33	287	449	949	1081.2794	Cy 3-caffeoyl-*p*-coumarylsoph-5glc
12b	8.35	287	433	919	1111.2898	Cy 3-caffeoyl-feruloylsoph-5glc
13a	8.52	301	463	907	1111.2898	Peo 3-dicaffeoylsoph-5glc
13b	8.62	301	463	949	1069.2791	Peo 3-caffeoyl-*p*-hydroxybenzoylsoph-5glc
14a	9.05	301	463	933	1095.2942	Peo 3-caffeoyl-*p*-coumarylsoph-5glc
14b	9.05	301	463	963	1125.3038	Peo 3-caffeoyl-feruloylsoph-5glc

^a^ Rt = retention time ^b^ Cy = cyanidin, Peo = peonidin, soph = sophorside, glc = glucoside.

**Table 2:** Anthocyanin monomer (1–14) content in cultivar Fushu No. 23 and cultivar Fushu No. 317 as determined by ultra performance liquid chromatography (μg/g fresh weight, FW).

Identity ^a^	Fushu No. 23			Fushu No. 317		
	Lf1	Lf2	Lf3	Lf1	Lf2	Lf3
Cy 3-soph-5glc	0.93 ± 0.22	1.65 ± 0.10	0.58 ± 0.06	3.47 ± 0.36	1.36 ± 0.17	0.21 ± 0.02
Peo 3-soph-5glc	0.68 ± 0.36	2.31 ± 0.12	1.40 ± 0.21	5.88 ± 0.84	2.76 ± 0.60	0.53 ± 0.05
Cy 3-*p*-hydroxybenzoylsoph-5glc	2.46 ± 0.50	6.66 ± 0.39	5.71 ± 0.43	10.41 ± 0.10	4.06 ± 1.11	0.63 ± 0.06
Cy 3-caffeylsophsoph-5glc	0.14 ± 0.00	0.40 ± 0.07	0.79 ± 0.11	11.09 ± 1.14	4.55 ± 1.08	0.31 ± 0.02
Peo 3-*p*-hydroxybenzoylsoph-5glc	0.98 ± 0.49	3.55 ± 0.11	2.85 ± 0.22	5.72 ± 0.05	2.76 ± 1.00	0.45 ± 0.08
Peo 3-caffeylsophsoph-5glc	0.02 ± 0.00	0.11 ± 0.02	0.18 ± 0.01	5.45 ± 1.16	2.00 ± 0.67	0.14 ± 0.01
Cy 3-*p*-coumarylsoph-5glc	0.06 ± 0.01	0.17 ± 0.04	0.12 ± 0.01	21.23 ± 2.04	7.42 ± 1.91	0.55 ± 0.05
Cy 3-feruloylsoph-5glc	0.21 ± 0.01	0.38 ± 0.03	0.45 ± 0.08	4.65 ± 0.72	1.63 ± 0.19	0.31 ± 0.05
Peo 3-*p*-coumarylsoph-5glc	0.11 ± 0.01	0.42 ± 0.07	0.31 ± 0.01	15.42 ± 0.79	6.84 ± 2.22	0.35 ± 0.04
Peo 3-feruloylsoph-5glc	0.05 ± 0.03	0.18 ± 0.04	0.20 ± 0.01	4.50 ± 0.85	1.45 ± 0.35	0.33 ± 0.06
Cy 3-dicaffeylsoph-5glc	0.24 ± 0.07	0.71 ± 0.13	3.27 ± 0.66	11.08 ± 0.82	6.34 ± 2.36	0.11 ± 0.01
Cy 3-caffeoyl-*p*-hydroxybenzoylsoph-5glc	1.43 ± 0.28	4.04 ± 0.61	8.57 ± 1.54	4.52 ± 0.40	2.61 ± 0.95	0.09 ± 0.01
Cy 3-caffeoyl-*p*-coumarylsoph-5glc	0.02 ± 0.00	0.10 ± 0.03	0.16 ± 0.04	7.24 ± 0.53	4.18±1.27	0.05 ± 0.00
Cy 3-caffeoyl-feruloylsoph-5glc	0.06 ± 0.03	0.21 ± 0.02	0.70 ± 0.22	5.07 ± 0.69	2.14 ± 0.40	0.10 ± 0.00
Peo 3-dicaffeoylsoph-5glc	0.02 ± 0.00	0.09 ± 0.01	0.40 ± 0.03	4.81 ± 0.35	2.72 ± 1.11	0.04 ± 0.00
Peo 3-caffeoyl-*p*-hydroxybenzoylsoph-5glc	0.20 ± 0.09	0.88 ± 0.08	1.56 ± 0.06	2.31 ± 0.14	1.42 ± 0.61	0.04 ± 0.00
Peo 3-caffeoyl-*p*-coumarylsoph-5glc	-	0.02 ± 0.00	0.02 ± 0.00	2.57 ± 0.28	2.12 ± 0.67	0.01 ± 0.00
Peo 3-caffeoyl-feruloylsoph-5glc	-	0.04 ± 0.01	0.14 ± 0.02	1.92 ± 0.45	0.85 ± 0.24	0.03 ± 0.00
Total anthocyanin	7.62 ± 1.43	21.92 ± 1.25	27.41 ± 2.69	127.33 ± 7.55	57.20 ± 6.75	4.28 ± 0.25
non-acylated anthocyanin	0.98 ± 0.24	1.84 ± 0.14	0.78 ± 0.06	7.97 ± 1.21	2.81 ± 0.51	0.53 ± 0.09
monoacylated anthocyanin	6.01 ± 1.75	17.98 ± 1.48	23.46 ± 3.42	60.99 ± 4.58	29.39 ± 8.78	2.28 ± 0.24
diacylated anthocyanin	0.63 ± 0.13	2.10 ± 0.25	3.17 ± 0.21	58.37 ± 5.93	25.00 ± 7.62	1.48 ± 0.17
cyanidin-based anthocyanin	5.59 ± 1.59	15.66 ± 0.93	12.38 ± 1.11	83.32 ± 7.19	33.37 ± 8.95	3.47 ± 0.38
peonidin-based anthocyanin	2.03 ± 0.52	6.26 ± 0.94	15.03 ± 2.58	44.01 ± 4.52	23.83 ± 7.96	0.81 ± 0.11

^a^ Cy = cyanidin, Peo = peonidin, soph = sophorside, glc = glucoside.

**Table 3:** Quantity of anthocyanins (1–14) from the leaves and roots of different sweet potato cultivars (μg/g FW).

Identity ^a^	Leaves					Roots				
	Fushu No. 23	Fushu No. 317	Fushu No. 25	OP	Yanshu No. 5	Fushu No. 9	Fushu No. 24	Longzishu No. 4	Longzishu No. 6	Fushu No. 317
Cy 3-soph-5glc	3.16 ± 0.27	5.04 ± 0.62	4.27 ± 0.32	7.51 ± 0.46	3.75 ± 0.26	2.82 ± 0.16	3.60 ± 0.25	1.30 ± 0.09	3.21 ± 0.32	42.02 ± 2.54
Peo 3-soph-5glc	4.38 ± 0.46	9.16 ± 0.94	2.62 ± 0.15	5.38 ± 0.32	8.15 ± 0.52	4.82 ± 0.25	4.02 ± 0.35	5.13 ± 0.69	27.41 ± 1.86	57.40 ± 3.68
Cy 3-*p*-hydroxybenzoylsoph-5glc	14.83 ± 1.98	15.09 ± 2.21	3.58 ± 0.16	2.55 ± 0.15	1.27 ± 0.08	2.87 ± 0.12	3.41 ± 0.27	3.24 ± 0.73	12.62 ± 0.79	112.39 ± 8.52
Cy 3-caffeylsophsoph-5glc	1.33 ± 0.15	15.94 ± 2.66	14.31 ± 0.86	16.64 ± 1.12	14.19 ± 1.03	1.72 ± 0.08	7.51 ± 0.43	16.15 ± 0.89	27.93 ± 1.56	19.97 ± 1.64
Peo 3-*p*-hydroxybenzoylsoph-5glc	7.39 ± 0.66	8.93 ± 1.04	5.10 ± 0.35	12.33 ± 0.84	6.17 ± 0.46	1.12 ± 0.07	13.84 ± 0.95	1.65 ± 0.06	4.91 ± 0.23	135.07 ± 9.36
Peo 3-caffeylsophsoph-5glc	0.31 ± 0.06	7.58 ± 0.98	3.70 ± 0.21	10.47 ± 0.76	6.75 ± 0.81	0.99 ± 0.11	0.77 ± 0.06	-	3.67 ± 0.28	13.28 ± 1.12
Cy 3-*p*-coumarylsoph-5glc	0.34 ± 0.07	29.20±1.45	22.26 ± 2.00	40.33 ± 2.38	20.16 ± 1.74	-	-	-	-	-
Cy 3-feruloylsoph-5glc	1.04 ± 0.15	6.59 ± 0.73	8.08 ± 0.19	9.10 ± 0.55	5.87 ± 0.44	11.46 ± 1.34	1.21 ± 0.08	8.53 ± 0.77	19.29 ± 1.64	11.32 ± 0.88
Peo 3-*p*-coumarylsoph-5glc	0.84 ± 0.11	22.61 ± 1.56	27.73 ± 1.87	31.23 ± 2.53	15.62 ± 1.15	-	4.15 ± 0.41	2.81 ± 0.16	8.47 ± 0.53	51.15 ± 3.63
Peo 3-feruloylsoph-5glc	0.43 ± 0.05	6.27 ± 0.42	7.69 ± 0.23	8.67 ± 0.79	5.58 ± 0.65	19.71 ± 1.74	3.47 ± 0.26	7.84 ± 0.63	61.44 ± 3.86	17.28 ± 1.41
Cy 3-dicaffeylsoph-5glc	4.23 ± 0.51	17.53 ± 2.08	21.49 ± 1.68	24.21 ± 1.82	12.1 ± 1.04	1.04 ± 0.07	3.11 ± 0.22	16.72 ± 0.92	14.07 ± 1.12	41.56 ± 3.76
Cy 3-caffeoyl-*p*-hydroxybenzoylsoph-5glc	14.04 ± 2.11	7.22 ± 0.55	8.85 ± 0.66	9.97 ± 0.73	6.42 ± 0.47	3.27 ± 0.19	-	3.04 ± 0.23	3.99 ± 0.24	32.23 ± 2.09
Cy 3-caffeoyl-*p*-coumarylsoph-5glc	0.28 ± 0.05	11.48 ± 0.92	14.07 ± 1.12	15.85 ± 1.75	7.92 ± 0.78	-	-	-	-	-
Cy 3-caffeoyl-feruloylsoph-5glc	0.97 ± 0.10	7.30 ± 0.67	5.06 ± 0.36	10.09 ± 0.95	6.50 ± 0.47	9.15 ± 1.22	0.91 ± 0.04	17.33 ± 0.81	3.77 ± 0.13	82.11 ± 5.49
Peo 3-dicaffeoylsoph-5glc	0.50 ± 0.07	7.58 ± 0.72	6.37 ± 0.43	10.47 ± 1.07	5.23 ± 0.32	13.07 ± 1.68	10.63 ± 0.78	40.75 ± 2.58	63.36 ± 4.75	37.02 ± 1.83
Peo 3-caffeoyl-*p*-hydroxybenzoylsoph-5glc	2.64 ± 0.33	3.77 ± 0.21	8.26 ± 0.61	5.21 ± 0.33	3.36 ± 0.23	1.52 ± 0.13	1.00 ± 0.03	18.17 ± 0.94	1.62 ± 0.08	166.51 ± 10.25
Peo 3-caffeoyl-*p*-coumarylsoph-5glc	0.04 ± 0.01	4.70 ± 0.37	5.76 ± 0.16	6.49 ± 0.49	3.25 ± 0.28	-	-	-	-	181.75 ± 9.58
Peo 3-caffeoyl-feruloylsoph-5glc	0.18 ± 0.02	2.80 ± 0.22	3.43 ± 0.04	3.87 ± 0.22	2.49 ± 0.17	3.75 ± 0.26	-	-	-	8.22 ± 0.63
Total anthocyanin	56.95 ± 7.10	188.81 ± 18.35	172.65 ± 11.41	230.36 ± 17.26	134.8 ± 9.65	77.32 ± 7.42	57.64 ± 4.13	142.66 ± 9.50	255.75 ± 17.39	1009.29 ± 66.41

^a^ Cy = cyanidin, Peo = peonidin, soph = sophorside, glc = glucoside.

## Supplementary Materials

The following are available online, Figure S1: UPLC-QTOF-MS/MS of anthocyanins in two purple sweetpotato leaves, Table S1: Primer sequences used for detection of genes related to anthocyanin biosynthesis by qRT-PCR.

## Abbreviations

PAL	phenylalanine ammonialyase
C4L	cinnamic acid-4-hydroxylase
4CL	4-coumarate:CoA ligase
CHS	chalconesynthase
CHI	chalconeisomerase
F3H	flavanone 3-hydroxylase
F3′H	flavonoids 3′-hydroxylase
DFR	dihydrofavonol 4-reductase
ANS	anthocyanin synthase
UFGT	UDP-glucose flavonoid 3-*O*-glucosyltransferase
3AT	anthocyanin 3-*O*-acyltransferases
qRT-PCR	quantitative real time polymerase chain reaction
SD	standard deviation
UPLC-QTOF-MS	ultra-performance liquid chromatography/quadrupole time-of-flight mass spectrometry
UPLC-DAD	ultra-performance liquid chromatography diode-array detection
FW	fresh weight
