# Supplementary material for: Anthocyanin Accumulation in the Leaves of the Purple Sweet Potato (Ipomoea batatas L.) Cultivars
Source: Molecules. 2019 Oct 17;24(20):3743. doi: 10.3390/molecules24203743 (PMC6832942; doi:10.3390/molecules24203743)
Supplement: Supplementary file 1 [file molecules-24-03743-s001.pdf]

Supplementary Information

Table 1. Primer sequences used for detection of genes related to anthocyanin biosynthesis by qRT-PCR.

Gene	Forward primer sequence (5'-3')	Reverse primer sequence (5'-3')
PAL	CATGGCGTCTATGGTTCTGT	TAAACTCCGGCTTCCCATTC
4CL	AACCAACCCGGAGAGATTTG	CAGCCATCCACGTCAATAGT
C4H	GTGGAGGAGGTGAAGAAGAATC	ACCTCCGGTCAAACATAATCC
CHS	GGGCTCACGTTCCATTTACT	GGAAATGCCGAGAGGATTGA
CHI	TTGAGAAGCTGACACGGATAAC	TCTGCATCGCCATACATTCC
DFR1	GCATGGAAAGCAGCCAAAG	CTGAGTGGGAATGTTGGTATGA
DFR2	GAAGCATGGAAAGCAGCTAAAG	ACTGGTGAGAGTGCGATAATG
DFR3	CATAGTGGAGGCAGGAAAGAAG	AGCAGAGATGGAGGAGGTAAA
DFR4	CTGCTGGAGTGATCTGGATTT	GTTGCTTTCCATGCTTCCTTC
F3H1	GTGACCATGGCCATCTCATTA	GACCTTGGTTGATCTCCTTCTC
F3H2	GGGTGTCTATAGCGACGTTTC	CGTGATGGGCTCTTCCATAAT
F3'H	GGGCGTTCGCAGAACTATTA	GGATTCTGTGACTAGCCGATTT
ANS	ACATAGCTGCAACAAGGGAATA	TCCAGCCTTCCTTCCTCTAA
UFGT1	TTCCGCCTCGCCAATAAA	TGAGGTGTGGGTGAAGATTG
UFGT2	CGACTCCCGTCAACCTTTATTC	TCTGGTAGTGAGGAGGAAGATG
UFGT3	GCCGGAGCTTGCTCTATTT	CCGATCGATACTTCTGACATTCC
UFGT4	CGATTCTGGAGGCGATATCTTT	TCCCATACACTCTCCACAAATC
3AT1-1	GGCGACGGGAGTAGTATATTTG	CGGAAAGTGAGAGGGATGAAG
3AT1-2	CGGCGGATAATGAACGGATAA	GGTGATGAGTGGTGCCAATA
3AT1-3	GTGGACTGCGCTTTCTATACTC	AGTCTTCAATGGCGGTTCTATC
3AT1-4	GTTCACCGCCTCATCTTCTATC	GTGTTGAAGGGTGAGGGATAAT
3AT1-5	CAGTTCACCGCCTCATCTT	GGCGAAGGGTGAGAGATAAAG
3AT1-6	GGCCTGGGAATTCTTCTCTAAT	GATTCCAGTCCTTTCGGATCTC
3AT1-7	CACCGCCTCATCTTCTATCAA	GAGGGTAGTGTTGAAGGGTAAG
3AT2	CCCTCACTCTAACTCGCATTTC	CGGCGAGAGGGAAATAATACTG
MYB1	GTGCTTAGGTGGTCGCTTAT	CATGGCAGACACCTTCTTCT
MYB60	GCGATCTGTTCCCACTAACA	GGGTAGCTCAAGTGGTAAGTAAG
MYB75-1	CAGCCGCTTCATCCGATAAT	GTCAATGGCGTCCGAGATAAT
MYB75-2	GCATGGTCGGAAGAAGAAGA	CACCTATTCAACCCAGCTCTAA
MYB113	CAACCTCCGATGAAGGAGATAAG	ACTGAATTCTCCCTCCTCAATAAA
ACT	CTTTGCCAAGAAGGAGATGC	TCTTGTCCTGACCACCAACA

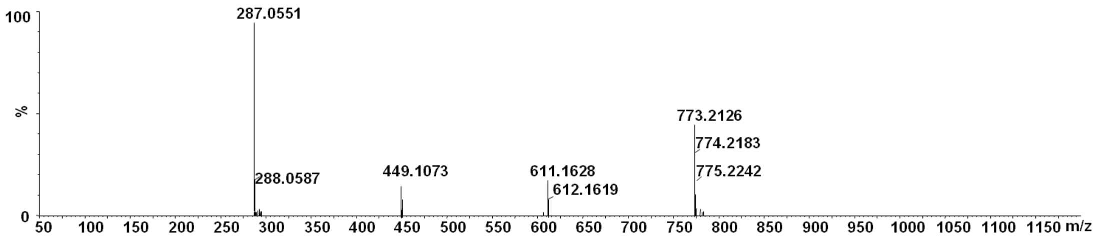

100
%
0
287.0551
288.0587
449.1073
611.1628
612.1619
773.2126
774.2183
775.2242
m/z

1

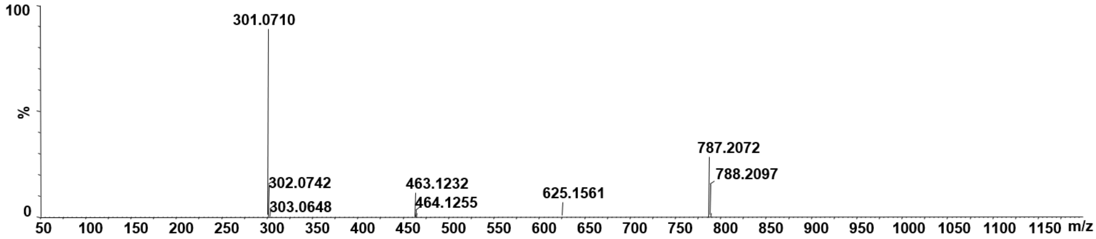

100
%
0
301.0710
302.0742
303.0648
463.1232
464.1255
625.1561
787.2072
788.2097
m/z

2

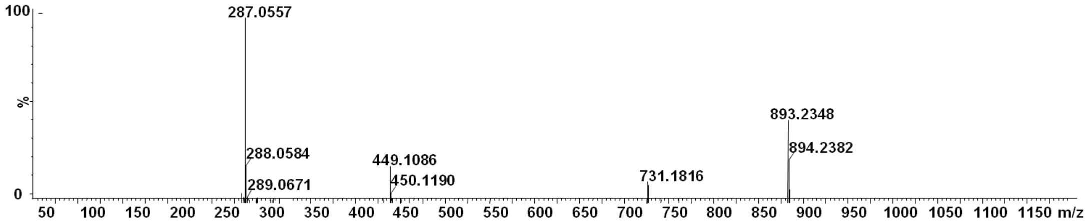

100
%
0
287.0557
288.0584
289.0671
449.1086
450.1190
731.1816
893.2348
894.2382
m/z

3

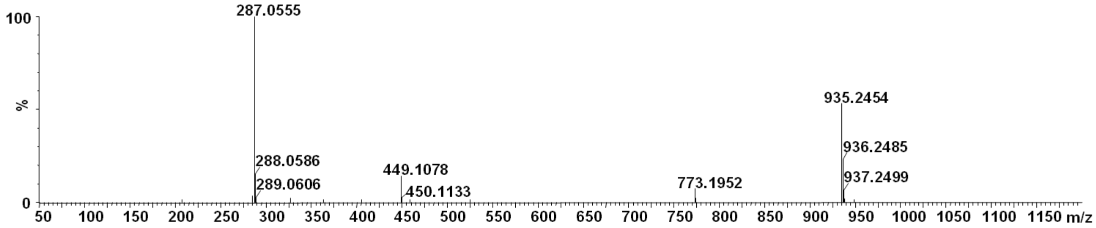

100
%
0
287.0555
288.0586
289.0606
449.1078
450.1133
773.1952
935.2454
936.2485
937.2499
m/z

4

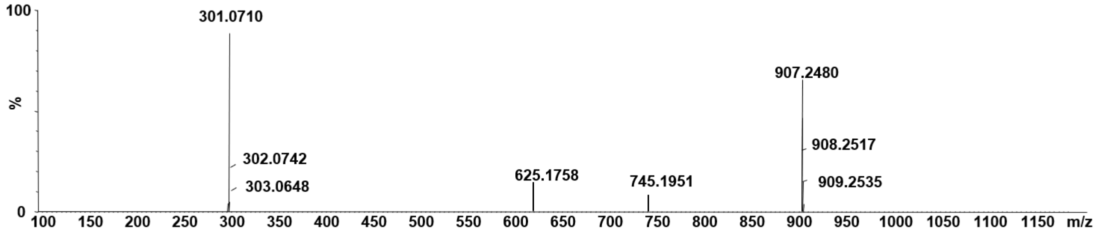

100
%
0
301.0710
302.0742
303.0648
625.1758
745.1951
907.2480
908.2517
909.2535
m/z

5

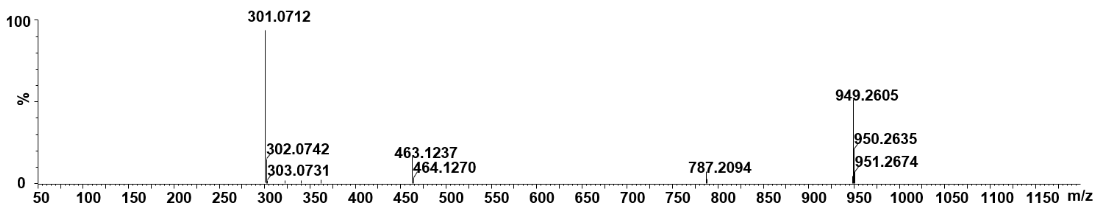

100
%
0
301.0712
302.0742
303.0731
463.1237
464.1270
787.2094
949.2605
950.2635
951.2674
m/z

6

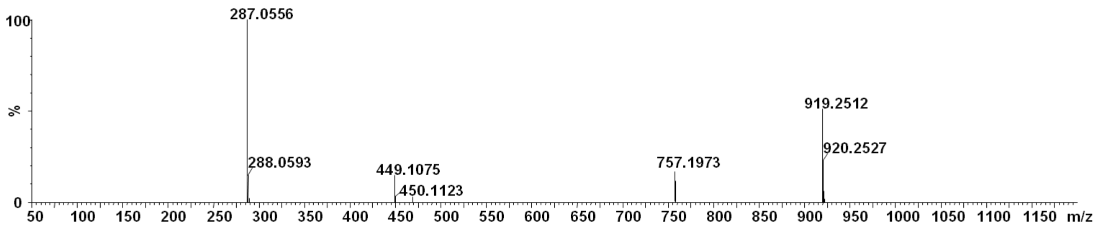

7

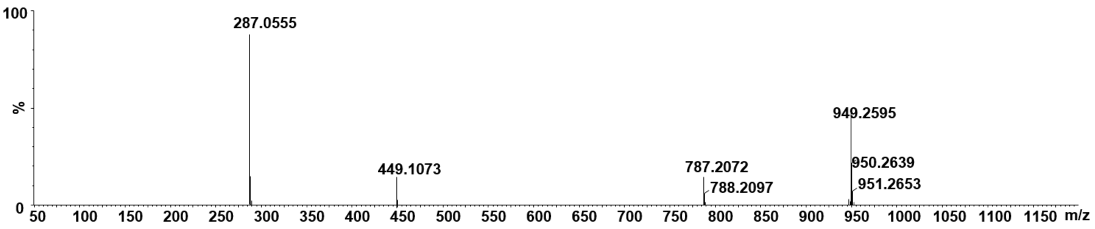

8

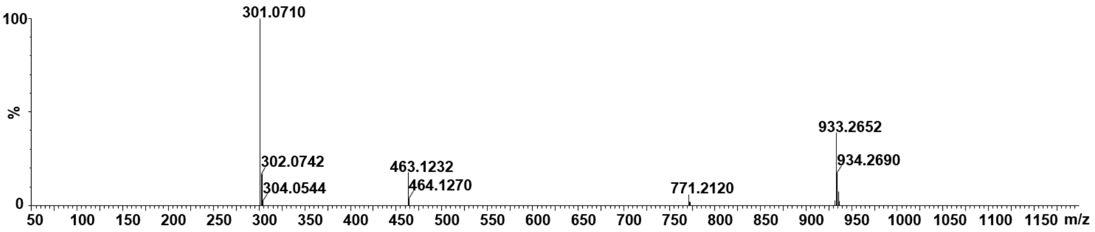

9

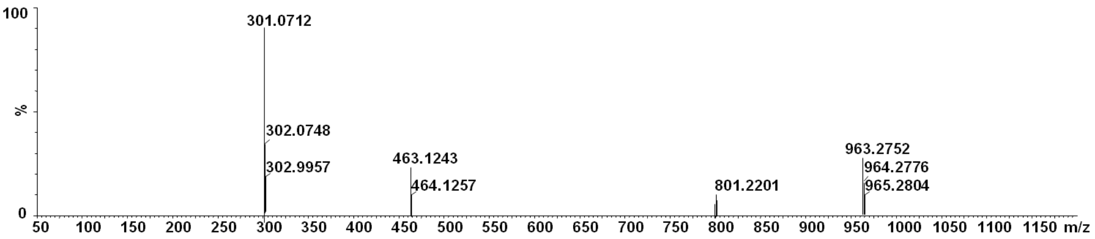

10

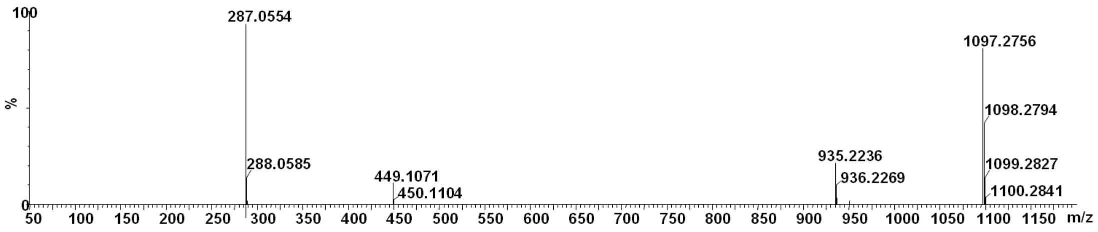

11a

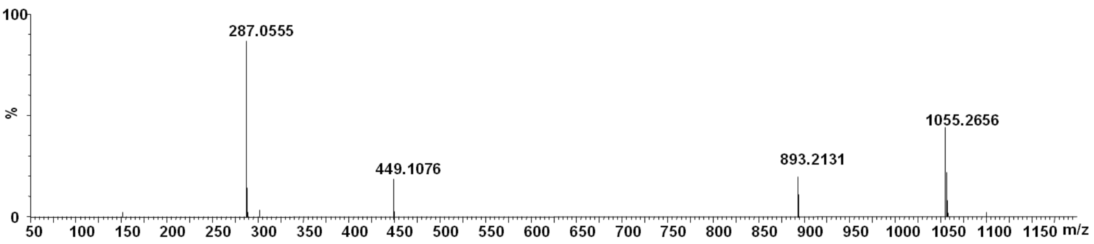

11b

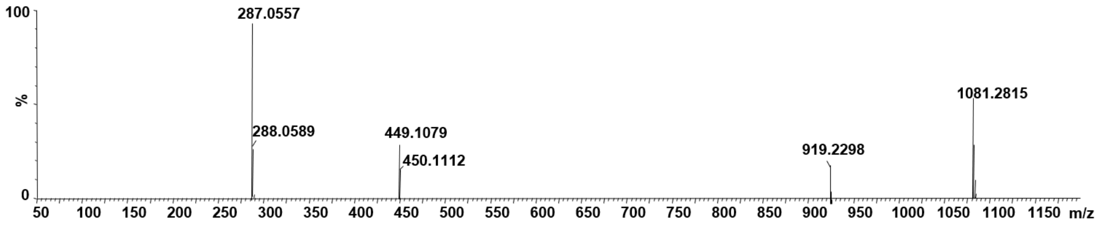

12a

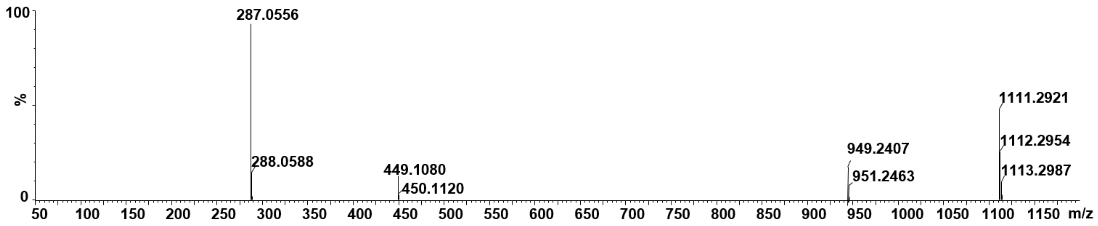

12b

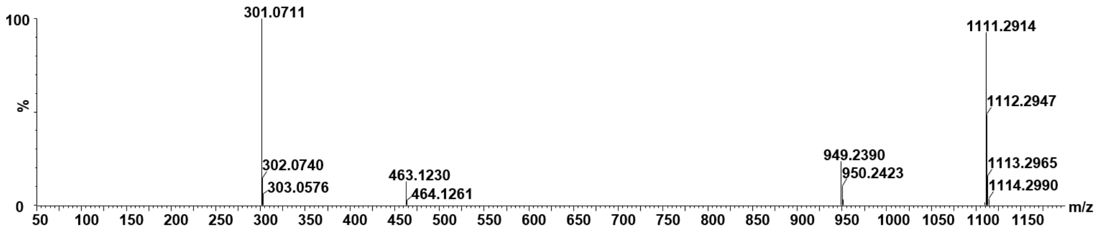

13a

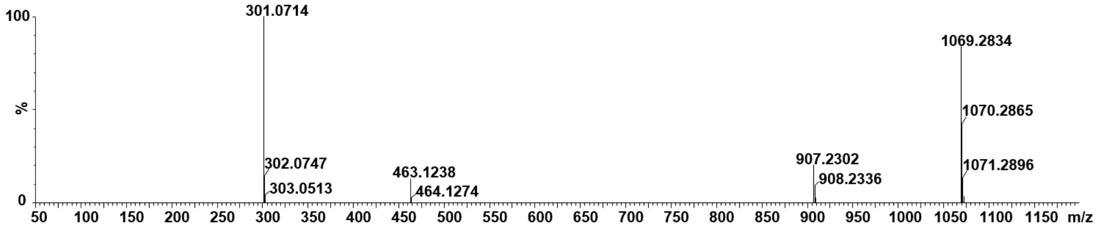

13b

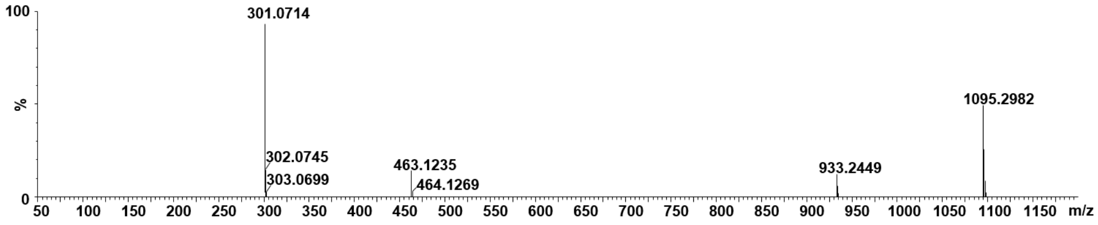

14a

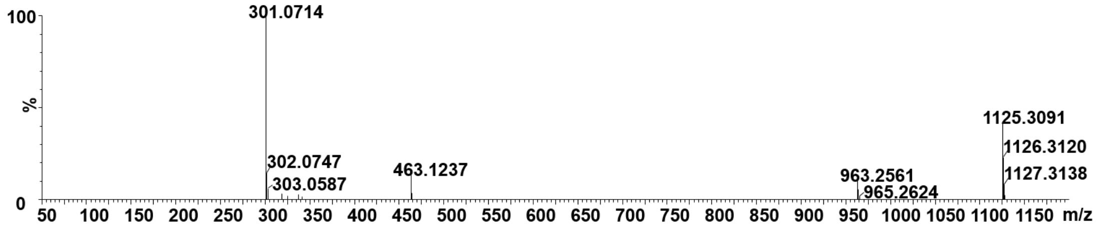

14b

Figure 1. UPLC-QTOF-MS/MS of anthocyanins in two purple sweetpotato leaves. Precursor analysis of (1) Cyanidin 3-sophoroside-5-glucoside; (2) Peonidin 3-sophoroside-5-glucoside; (3) Cyanidin

3-(6''-p-hydroxybenzoylsoph)-5-glucoside; (4) Cyanidin 3-(6''-caffeylsophoroside)-5-glucoside; (5) Peonidin 3-(6''-p-hydroxybenzoylsophoroside)-5-glucoside; (6) Peonidin 3-(6''-caffeoylsophoroside)-5-glucoside; (7) Cyanidin 3-(6''-p-coumarylsophoroside)-5-glucoside; (8) Cyanidin 3-(6''-feruloylsophoroside)-5-glucoside; (9) Peonidin 3-(6''-p-coumarylsophoroside)-5-glucoside; (10) Peonidin 3-(6''-feruloylsophoroside)-5-glucoside; (11a) Cyanidin 3-(6',6''-dicaffeylsophoroside)-5-glucoside; (11b) Cyanidin 3-(6'-caffeoyl-6''-p-hydroxybenzoylsophoroside)-5-glucoside; (12a) Cyanidin 3-(6'-caffeoyl-6''-feruloylsophoroside)-5-glucoside; (12b) Cyanidin 3-(6'-caffeoyl-6''-p-coumaryl sophoroside)-5-glucoside; (13a)Peonidin 3-(6',6''-dicaffeoylsophoroside)-5-glucoside; (13b) Peonidin 3-(6'-caffeoyl-6''-p-hydroxybenzoylsophoroside)-5-glucoside; (14a) Peonidin 3-(6'-caffeoyl-6''-p-coumarylsophoroside)-5-glucoside; (14b) Peonidin 3-(6'-caffeoyl-6''-feruloylsophoroside)-5-glucoside.
